# Licochalcones extracted from *Glycyrrhiza inflata* inhibit platelet aggregation accompanied by inhibition of COX-1 activity

**DOI:** 10.1371/journal.pone.0173628

**Published:** 2017-03-10

**Authors:** Asa Okuda-Tanino, Daiki Sugawara, Takumi Tashiro, Masaya Iwashita, Yutaro Obara, Takahiro Moriya, Chisato Tsushima, Daisuke Saigusa, Yoshihisa Tomioka, Kuniaki Ishii, Norimichi Nakahata

**Affiliations:** 1 Department of Cellular Signaling, Graduate School of Pharmaceutical Sciences, Tohoku University, Sendai, Japan; 2 Department of Oncology, Pharmacy Practice and Sciences, Graduate School of Pharmaceutical Sciences, Tohoku University, Sendai, Japan; 3 Department of Pharmacology, Yamagata University School of Medicine, Yamagata, Japan; Boston University, UNITED STATES

## Abstract

Licochalcones extracted from *Glycyrrhiza inflata* are known to have a variety of biological properties such as anti-inflammatory, anti-bacterial, and anti-tumor activities, but their action on platelet aggregation has not yet been reported. Therefore, in this study we investigated the effects of licochalcones on platelet aggregation. Collagen and U46619, a thromboxane A_2_ receptor agonist, caused rabbit platelet aggregation, which was reversed by pretreatment with licochalcones A, C and D in concentration-dependent manners. Among these compounds, licochalcone A caused the most potent inhibitory effect on collagen-induced platelet aggregation. However, the licochalcones showed marginal inhibitory effects on thrombin or ADP-induced platelet aggregation. In addition to rabbit platelets, licochalcone A attenuated collagen-induced aggregation in human platelets. Because licochalcone A also inhibited arachidonic acid-induced platelet aggregation and production of thromboxane A_2_ induced by collagen in intact platelets, we further examined the direct interaction of licochalcone A with cyclooxygenase (COX)-1. As expected, licochalcone A caused an inhibitory effect on both COX-1 and COX-2 *in vitro*. Regarding the effect of licochalcone A on COX-1 enzyme reaction kinetics, although licochalcone A showed a stronger inhibition of prostaglandin E_2_ synthesis induced by lower concentrations of arachidonic acid, Vmax values in the presence or absence of licochalcone A were comparable, suggesting that it competes with arachidonic acid at the same binding site on COX-1. These results suggest that licochalcones inhibit collagen-induced platelet aggregation accompanied by inhibition of COX-1 activity.

## Introduction

Platelets play critical roles in homeostatic functions through their adhesion and aggregation characteristics. When vessel walls are injured, platelets activate immediately to form a plug, and consequently blood coagulation and thrombi occlude the site of injury. A series of such responses is important for preventing vital blood loss, but it is possible that excessive enhancement of platelet function can lead to ischemia and destruction in vital organs.

Collagen is the most thrombogenic component of the subendothelium. Collagen binds to two receptors on platelets: integrin α_2_β_1_ promotes adhesion of platelets to collagen and permits them to interact with glycoprotein VI (GP VI) [1–3]. Then, stimulation of GP VI with collagen causes splicing of arachidonic acid at the inner plasma membranes of platelets by activating phospholipase A_2_ (PLA_2_) [4]. The released arachidonic acid is metabolized to thromboxane A_2_ (TXA_2_) by cyclooxygenase (COX)-1 and thromboxane synthase (TXS), whose chemical half-time is approximately 30 s. TXA_2_ released from activated platelets binds to TXA_2_ receptors to cause platelet morphological changes and aggregation as a positive feedback mediator [5, 6]. Representative anti-platelet agents presently used in the clinic are aspirin and ozagrel, which inhibit TXA_2_ generation [6], and sarpogrelate and clopidogrel, which are receptor antagonists of 5-hydroxy tryptophan and ADP, respectively [7, 8].

Licochalcones are purified from the licorice species *Glycyrrhiza inflata*, a well-known herb, and are usually contained in Chinese medicines. They have been shown to have a variety of biological properties, such as anti-inflammatory, anti-bacterial, anti-tumor, anti-diabetic activities [9–13]. In addition, it has been reported that glycyrrhizin, which is also a major component of *Glycyrrhiza inflata*, binds to thrombin and inhibits platelet aggregation induced by thrombin with an IC_50_ of approximately 150 μM [14]. In the present study, we examined the effects of licochalcones on rabbit platelets and human platelets, and demonstrated that licochalcones inhibited platelet aggregation induced by collagen at lower concentrations compared with that by U46619, and was accompanied by an interaction with COX-1.

## Materials and methods

### Materials

Licochalcones were provided by Research Laboratory of Minophagen Pharmaceutical Co. Ltd. (Zama, Japan). Prostaglandin (PG) E_2_, *d*_4_-PGE_2_, U46619, COX-1, COX-2 and TXB_2_ EIA Kit were purchased from Cayman Chemical Company (Ann Arbor, MI). Collagen (Collagenreagent Horm) was purchased from Nycomed Pharma GMBH (Marburg, Germany). Thrombin was purchased from Wako Pure Chemicals (Osaka, Japan). Arachidonic acid and ADP were purchased from Sigma-Aldrich (St. Louis, MO) or Cayman Chemical Company. All other chemicals used were of reagent grade or the highest quality available.

### Preparation of washed platelets

Washed platelets were prepared from healthy male rabbits (Japanese white rabbits weighing approximately 2.5–3.5 kg), and were collected in plastic tubes containing acid citrate dextrose solution (1/6 volume of blood) composed of citric acid (65 mM), trisodium citrate (85 mM), and D-glucose (2%). Then, the blood was centrifuged at 250 × *g* for 12 min to obtain platelet-rich plasma. The platelet-rich plasma was centrifuged at 90 × *g* to remove contaminated erythrocytes and leukocytes, and then centrifuged at 450 × *g* for 15 min at room temperature (20–25°C). The pellet was washed twice with Tyrode/HEPES solution (NaCl 138.3 mM, KCl 2.68 mM, MgCl_2_·6H_2_O 1.0 mM, NaHCO_3_ 4.0 mM, HEPES 10 mM, glucose 0.1%, and bovine serum albumin 0.35% at pH 6.35). The resultant pellet was suspended in the second Tyrode/HEPES solution (pH 7.35) with a final density of 3–5×10^8^ platelets/ml [15]. Human washed platelets collected from volunteers were prepared similarly. The present study using human platelets was performed in accordance with a protocol approved by the Ethics Committee of Graduate School of Pharmaceutical Sciences, Tohoku University (approval No.12-04). Informed consent was obtained in written forms with volunteers’ signatures. The present study using rabbit platelets was performed in accordance with a protocol approved by the Institutional Animal Care and Use Committee of the Tohoku University Environmental and Safety Committee (approval No. 22Yaku-Do-29).

### Determination of platelet aggregation

Platelet aggregation was determined by a standard turbidimetric method using an aggregometer (PAM-6C, Merbanix, Tokyo, Japan), as described previously [15, 16]. Platelet aggregation was expressed as an increase in light transmission. The levels of light transmission were calibrated as 0% for a platelet suspension and 100% for the Tyrode/HEPES solution (pH 7.35). Platelet suspension (3×10^8^ platelets/ml in 0.3 ml) in a cuvette was preincubated at 37°C for 2–3 min under continuous stirring at 1000 rpm. CaCl_2_ was then added at a final concentration of 1 mM for 3 min. After the pre-incubation of licochalcones for 5 min, platelet aggregation was initiated by the addition of U46619, collagen, thrombin, ADP or arachidonic acid and monitored for 10 min. Maximal platelet aggregation was achieved at the applied concentration of these stimulators.

### Observation of platelets by scanning electron microscopy

Samples for observation by scanning electron microscopy were prepared as described previously [16]. Briefly, washed platelet aggregation was initiated by collagen stimulation for 3 min in the presence or absence of licochalcones, and then fixed overnight with 1% glutaraldehyde. Samples were washed twice with phosphate-buffered saline (PBS) for 5 min. The fixed platelets were dehydrated with increasing concentrations of ethanol (50, 70, 80, 90, and 100%) and t-butyl alcohol. The samples were then freeze-dried (ES-2030, Hitachi, Tokyo, Japan) and sputter-coated with Au/Pd with an ion sputter (E-1010, Hitachi, Tokyo, Japan). These samples were observed with a scanning electron microscope (S-3200, Hitachi, Tokyo, Japan).

### Measurement of TXB_2_

Quantification of TXB_2_ derived from platelets was performed using a TXB_2_ EIA kit. Washed platelets were preincubated at 37°C for 3 min under continuous stirring at 1000 rpm. CaCl_2_ (1 mM) was then added for 3 min. After the pre-incubation with licochalcone A for 5 min, platelet aggregation was initiated by addition of collagen (3 μg/ml) and the reactions were stopped by indomethacin/EDTA (25 μM/25 mM) at 4°C. Samples were centrifuged at 2500 × *g* for 3 min, and then the supernatant was measured by EIA. EIA procedures were performed as indicated in the assay kit instructions.

### Determination of COX-1 and COX-2 activity by LC-MS/MS

COX inhibition assays were carried out following with modifications of previous report [17]. A 100 mM Tris-HCl buffer (pH 8.0, 222 μL) containing 2 μM hematin and 5 mM L-tryptophan was added to a 2 mL tube. Then, two units of COX-1 or COX-2 (2.5 μL) in Tris-HCl buffer (pH 8.0) and each concentration of the licochalcone A (0–100 μM, 1.25 μL) in DMSO were added to the assay for determining IC_50_ value, and the sample was incubated at 37°C for 10 min. The reaction was initiated by adding 0.5 μM arachidonic acid (25 μL) in Tris-HCl buffer (pH 8.0). After 2 min, the reaction was terminated by adding 1.0 M HCl (12.5 μL). Then, methanol (5 μL) which included internal standard (IS) (*d*_4_-PGE_2_, 50 ng/mL) to calculate the area ratio (peak area of PGE_2_/*d*_*4*_-PGE_2_) of compounds for calibration, was added to the sample. After 30 min, PGE_2_ and IS were extracted by hexane/ethyl acetate (800 μL, 50/50, v/v%). The sample was mixed and centrifuged at 11,042 × *g* for 5 min at 4°C, and the organic supernatant was transferred to another tube. Then, the sample was dried under vacuum. Finally, the sample was dissolved in acetonitrile/water (50 μL, 50/50, v/v%) containing 0.1% formic acid, and filtered (0.2 μm pore size, acetyl cellulose, YMC). Five microliters of sample was subjected to LC-MS/MS.

### Enzyme kinetics analysis of COX-1

A 100 mM Tris-HCl buffer (pH 8.0, 222 μL) containing 2 μM hematin and 5 mM L-tryptophan was added to a 2 mL tube. Then, two units of COX-1 (2.5 μL) in Tris-HCl buffer (pH 8.0) and 2.5 μM licochalcone A (1.25 μL) in DMSO were added, and the sample was incubated at 37°C for 10 min. The reaction was initiated by adding each concentration of arachidonic acid (0.1–10 μM in 25 μL) in Tris-HCl buffer (pH 8.0). After 2 min, the reaction was terminated by adding 12.5 μL of 1.0 M HCl, and the samples for LC-MS/MS were prepared as described in above section.

### LC-MS/MS analysis

The HPLC separations were performed on a Nanospace SI-2 HPLC system (Shiseido, Tokyo) using an XBridge (Waters Corp., Milford, MA) C18 analytical column (50 mm × 2.1 mm i.d., 3.5 μm particle size) with an isocratic elution with a mobile phase consisting of acetonitrile/water (32/68, v/v %) containing 0.1% formic acid. The column oven was maintained at 40°C, and the flow rate was 200 μL/min. The HPLC system was coupled with a Fourier transform mass spectrometer (FTMS, QExactive, Thermo Fisher Scientific, San Jose, CA) equipped with a heated ESI (HESI) source. The ion source parameters were as follows: spray voltage, 3,000 V (negative ion mode); capillary and heater temperature, 250°C and 250°C; sheath gas pressure (nitrogen), 40 psi; auxiliary gas pressure (nitrogen), 5 psi; S-lens RF level, 50%. Samples were analyzed in the targeted-MS^2^ (t-MS^2^) mode. The t-MS^2^ settings were as follows: resolution, 17,500; auto gain control target, 2E5; isolation width, 2.0 m/z; collision energy, 25% for PGE_2_ and *d*_*4*_-PGE_2_. The inclusion list was *m/z* = 351.21725 for PGE_2_, and 355.24226 for *d*_4_-PGE_2_. The product ion was *m/z* = 271.20691 for PGE_2_, and 275.23206 for *d*_4_-PGE_2_.

### Data analysis

Data are expressed as mean±S.E.M. Significant differences were analyzed using Student’s t-test or Tukey–Kramer’s and Dunnett’s methods for multiple comparison.

## Results

### Licochalcones inhibit platelet aggregation

**Fig 1:**
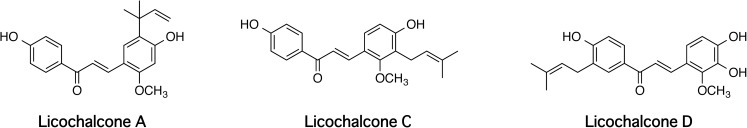
Chemical structures of licochalcones A, C and D.

**Fig 2:**
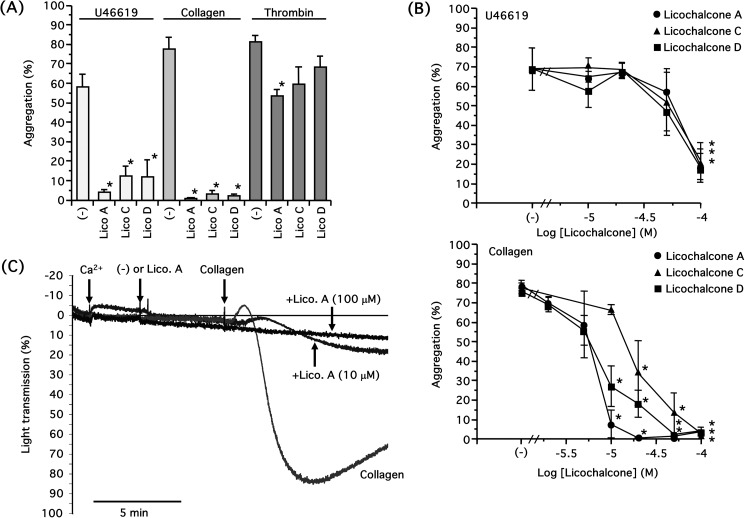
Concentration-dependent inhibition of U46619- and collagen-induced platelet aggregation by licochalcones. **(A)** Licochalcone A (Lico A, 100 μM), licochalcone C (Lico C, 100 μM), licochalcone D (Lico D, 100 μM) or DMSO (-) was preincubated for 5 min before addition of U46619 (3 μM), collagen (3 μg/ml) or thrombin (0.03 U/ml) in the presence of 1 mM CaCl_2_. Results are shown as mean±S.E.M. (*P<0.05 compared with control, n = 3, Tukey–Kramer’s method). **(B)** Licochalcones (2–100 μM) or DMSO (control) were preincubated for 5 min before addition of U46619 (3 μM) or collagen (3 μg/ml) in the presence of 1 mM CaCl_2_. Results are shown as mean±S.E.M. (*P<0.05 compared with control, n = 3–8, Dunnett’s method). **(C)** Licochalcone A (10 or 100 μM) or DMSO (control) were preincubated for 5 min before addition of collagen (3 μg/ml) in the presence of 1 mM CaCl_2_. Representative traces of the collagen-induced platelet aggregation with or without licochalcone A are shown.

**Fig 3:**
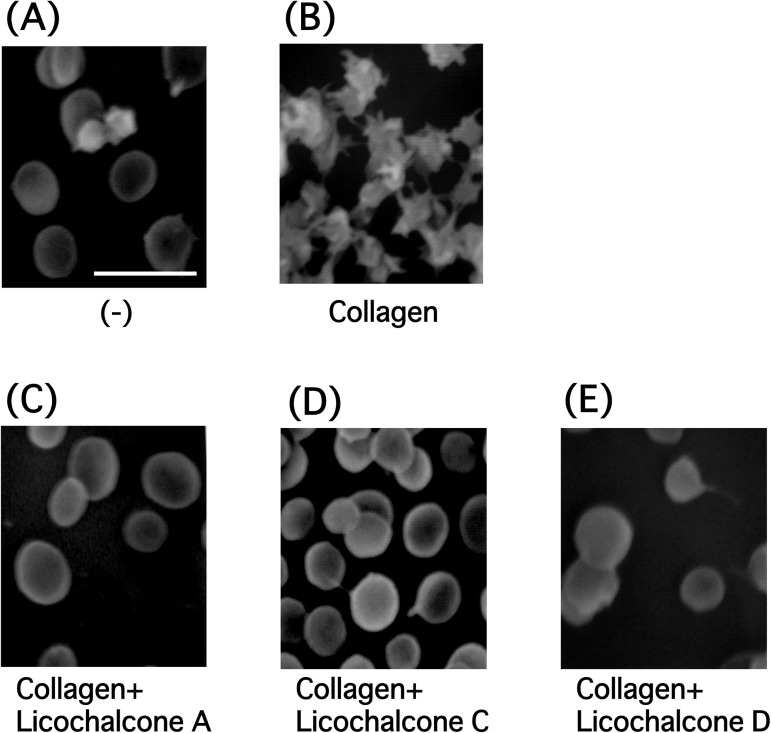
Scanning electron microscopy images of platelets stimulated by collagen in the presence of licochalcones. Platelets were incubated with collagen (3 μg/ml) for 3 min in the presence or absence of licochalcones (100 μM), then fixed overnight with 1% glutaraldehyde. The samples were washed twice for 5 min with PBS. The fixed platelets were dehydrated with ethanol and t-butyl alcohol, and after the samples were freeze-dried and coated with Au/Pd, they were observed under a scanning electron microscope. **(A)** Unstimulated platelets, **(B)** collagen (3 μg/ml), **(C)** licochalcone A (100 μM) +collagen, **(D)** licochalcone C (100 μM) +collagen and **(E)** licochalcone D (100 μM) +collagen. (Magnification: 7000×, bar = 5 μm).

**Fig 4:**
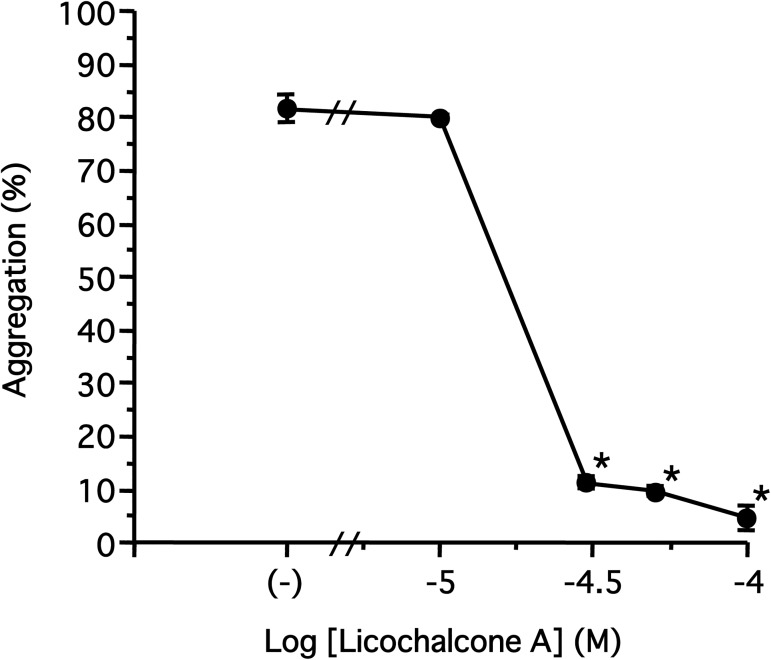
Concentration-dependent inhibition of collagen-induced human platelet aggregation by licochalcone A. Licochalcone A (10–100 μM) or DMSO (control) were preincubated for 5 min before addition of collagen (5 μg/ml) in the presence of 1 mM CaCl_2_. Results are shown as mean±S.E.M. (*P<0.05 compared with control, n = 3, Dunnett’s method).

We first examined the effects of licochalcones on platelet aggregation. There are several molecular species of licochalcones (Fig 1); we used licochalcone A, licochalcone C, and licochalcone D at a concentration of 100 μM. All licochalcones significantly inhibited U46619 (3 μM)- and collagen (3 μg/ml)-induced platelet aggregation in concentration-dependent manners, and collagen-induced platelet aggregation was particularly impaired compared with that induced by U46619 (Fig 2). However, the inhibition of thrombin (0.03 U/ml) or ADP (10 μM)-induced aggregation by licochalcones (100 μM) was marginal (Fig 2A and S1 Fig). Additionally, licochalcone A was more effective in inhibiting platelet aggregation than the other licochalcones. Licochalcone A inhibited the collagen-induced platelet aggregation with an IC_50_ value of approximately 6.33 μM, whereas licochalcone C was 17.6 μM and licochalcone D was 7.58 μM (Fig 2). We observed the morphology of platelets that aggregated by collagen in the presence of licochalcones with scanning electron microscopy. Platelets underwent morphological changes when they were stimulated by collagen; however, the platelets did not show any change following pretreatment with licochalcones (Fig 3). We further examined the inhibitory effect of licochalcone A on human platelet aggregation; licochalcone A also inhibited collagen-induced human platelet aggregation in a concentration-dependent manner (Fig 4).

### Licochalcone A attenuates collagen-induced platelet aggregation accompanied by inhibition of COX-1 activity

**Fig 5:**
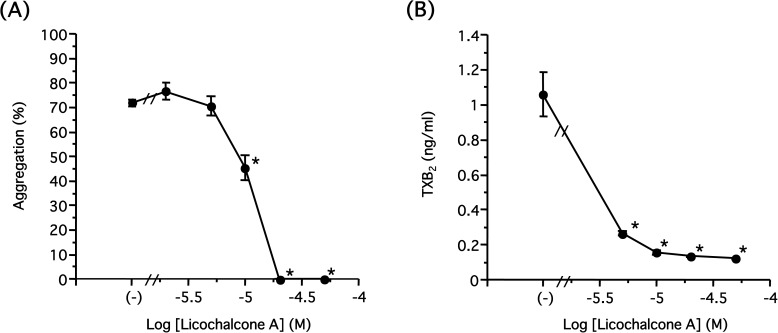
Concentration-dependent inhibition of arachidonic acid metabolism by licochalcone A. **(A)** Licochalcone A (2–50 μM) or DMSO (−) was preincubated for 5 min before addition of arachidonic acid (30 μM) in the presence of 1 mM CaCl_2_. Results are shown as mean±S.E.M. (*P<0.05 compared with control, n = 3, Dunnett’s method). **(B)** Licochalcone A (5–50 μM) or DMSO (−) was preincubated for 5 min before addition of collagen (3 μg/ml) in the presence of 1 mM CaCl_2_, and terminated by EDTA/indomethacin after incubating for 5 min. Samples were diluted 1/1000 and TXB_2_ amount was measured by EIA. Results are shown as mean±S.E.M. (*P<0.05 compared with control, n = 3, Dunnett’s method).

It is well known that collagen causes platelet aggregation mediated through arachidonic acid release by PLA_2_ at the inner plasma membrane of platelets, and COX inhibitors such as aspirin can block the platelet aggregation induced by collagen [16]. Therefore, we examined whether licochalcone A, which inhibited collagen-induced platelet aggregation at a lower concentration compared with the other licochalcones, also inhibits extrinsic arachidonic acid-induced platelet aggregation. Extrinsic arachidonic acid behaves like internal arachidonic acid because of its lipophilicity. Licochalcone A (˃20 μM) completely inhibited platelet aggregation induced by arachidonic acid (30 μM) (Fig 5A). These results suggest that licochalcone A affects molecules downstream of arachidonic acid, possibly COX-1 or TXS, for example.

**Fig 6:**
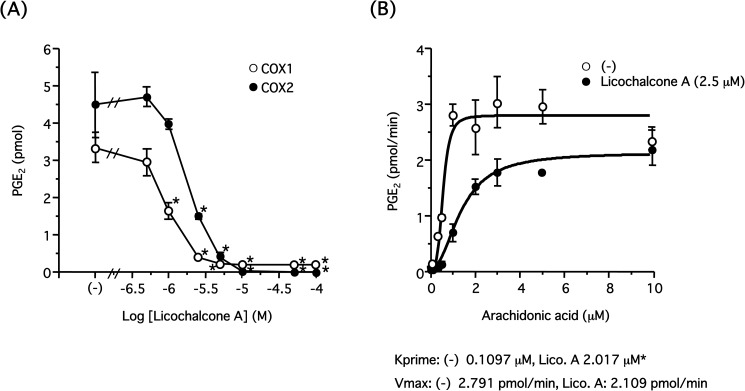
Competitive inhibition of COX-1 and COX-2 activity by licochalcone A *in vitro*. **(A)** Hematin (2 μM) and L-tryptophan (5 mM) were added to 100 mM Tris-HCl buffer (pH 8.0), then two units of COX-1 or COX-2 and each concentration of licochalcone A (0.5–100 μM) were added and incubated at 37°C for 10 min. The reaction was initiated by adding arachidonic acid (0.5 μM). After 2 min, the reaction was terminated by adding 1.0 M HCl, and then the amount of PGE_2_ was measured by LC-MS/MS. Results are shown as mean±S.E.M. (*P<0.05 compared with control, n = 3, Dunnett’s method). **(B)** Two units of COX-1 and licochalcone A (2.5 μM) were incubated at 37°C for 10 min as described above. The reaction was initiated by adding each concentration of arachidonic acid (0.1–10 μM). After 2 min, the reaction was terminated by adding 1.0 M HCl, and then the amount of PGE_2_ was measured by LC-MS/MS. Results are shown as mean±S.E.M. (*P<0.05 compared with control, n = 3, Student’s t-test).

Arachidonic acid released from the inner membrane interacts with COX-1 and is converted to PGH_2_, then is sequentially metabolized to prostaglandins including TXA_2_ and PGE_2_. Therefore, we examined the amount of TXB_2_, a metabolite of TXA_2_, released from platelets by collagen (3 μg/ml). As observed in Fig 5B, licochalcone A reduced the production of TXB_2_ in a concentration-dependent manner with an IC_50_ value of approximately 2.04 μM, suggesting licochalcone A inhibits COX-1 activity. Hence, we determined whether licochalcone A directly inhibited COX-1 and COX-2 *in vitro*. After COX-1 or COX-2 was preincubated in the presence of licochalcone A (0.5–100 μM), the enzyme reaction was initiated by the addition of arachidonic acid (0.5 μM), and newly synthesized PGE_2_ was measured by LC-MS/MS, as described in the Materials and Methods (Fig 6A). Licochalcone A reduced PGE_2_ production in a concentration-dependent manner with IC_50_ values of 0.94 and 1.93 μM for COX-1 and COX-2, respectively, indicating licochalcone A directly inhibited COX-1 and COX-2. Finally, to assess the inhibitory effect of licochalcone A on COX-1 activity, we also investigated the enzyme reaction kinetics. COX-1 (2 units) was preincubated in the presence or absence of licochalcone A (2.5 μM), then the reaction was started by the addition of arachidonic acid (0.1–10 μM). Newly synthesized PGE_2_ was measured by LC-MS/MS, as shown in Fig 6A (Fig 6B). In the case of the control (without licochalcone A), the values followed the Michaelis–Menten equation (Km 0.6236 μM, Vmax 3.213 pmol/min). However, in the presence of licochalcone A, PGE_2_ production resulting from lower concentrations of arachidonic acid was largely suppressed and showed a sigmoid curve. Hence, kinetics parameters were obtained by fitting the values to the sigmoid curve rather than the Michaelis–Menten equation. In this case, Vmax values for vehicle (-) and licochalcone A were 2.791 pmol/min and 2.109 pmol/min, respectively, and Kprime values (concentration corresponding to 1/2 Vmax) were 0.1097 μM and 2.017 μM. Vmax values were not significant and comparable regardless of licochalcone A, and Kprime values were significantly distinct following the addition of licochalcone A, suggesting that licochalcone A competitively inhibits COX-1 activity with arachidonic acid.

## Discussion

In the present study, we investigated the effects of licochalcones, which are contained in licorice and are frequently used in Chinese medicine, on human and rabbit platelet aggregation. The species of licorice used in the present study are more abundant in licochalcones than other species. First, we examined the effects of licochalcones on rabbit washed platelet aggregation. All licochalcones largely inhibited platelet aggregation induced by both U46619 and collagen, but their inhibitory effects on collagen-induced platelet aggregation were 10-fold more effective than that induced by U46619 with regards to IC_50_ (Fig 2). Therefore, we supposed that licochalcones acted more specifically on the aggregation induced by collagen, and licochalcone A was the most effective. Importantly, licochalcone A suppressed human platelet aggregation in addition to rabbit platelets (Fig 4). Furthermore, licochalcone A suppressed arachidonic acid-induced aggregation in a concentration-dependent manner, as in the case of collagen-induced aggregation (Fig 5A); therefore, we postulated that licochalcone A acted on COX-1 or TXS, but not on release of arachidonic acid from plasma membranes. Actually, we observed that licochalcone A had a direct inhibitory action on COX-1 *in vitro* (Fig 6) and inhibited production of TXA_2_ in intact platelets in a similar concentration range (Fig 5B). In addition, this range of concentration-dependency (IC_50_ 2.04 μM) (Fig 5B) is also almost comparable with that of platelet aggregation induced by collagen (3 μg/ml) (IC_50_ 6.33 μM) (Fig 2B). Although there is a slight difference about these IC_50_ values, we consider this results from the different methodology of these assays, i.e. we measured the accumulation of TXB_2_ for 5 min, whereas the platelet aggregation reflected the unstable TXA_2_ levels induced by collagen present at each time points.

These results strongly suggest that licochalcone A inhibits platelet aggregation by suppressing COX-1 activity. However, licochalcone A also inhibited the platelet aggregation induced by U46619 at higher concentrations (Fig 2). COX inhibitors generally do not suppress platelet aggregation induced by U46619 [18, 19], so this result suggests the possibility that licochalcones also act on molecules other than COX-1, with lower affinity in platelets. For example, licochalcones at higher concentration may act on TP directly as TP antagonists. As it has been reported that licochalcone A interacts with inhibitor-κB kinase (IKK) [20], it is necessary to identify the other molecules interacting with licochalcone A.

It has been demonstrated that the thrombin-induced platelet aggregation does not require TXA_2_ production through COX. Thus, COX inhibitors do not show inhibitory effects on the aggregation by thrombin as in case of U46119 [21, 22]. In case of thrombin, intracellular Ca^2+^ increase via protease-activated receptors (thrombin receptors)/G_q_/PLC-β pathway is essential. Hence, we assume that licochalcones did not inhibit the thrombin-induced aggregation efficiently (Fig 2). In addition, licochalcone A did not block the ADP-induced platelet aggregation (S1 Fig) as it has been shown that the ADP-induced platelet aggregation does not require COX activity [23, 24]. On the other hand, platelet aggregation by collagen requires COX activity, we and another group showed that NSAIDs such as aspirin and indomethacin completely blocked the platelet aggregation by collagen [16, 22]. In this study, we clarified that licochalcones were COX inhibitors, this is the reason to explain the different results between collagen and thrombin or ADP.

It has been shown that COX-1 isoforms predominantly localize at intracellular membranes including the endoplasmic reticulum, nuclear envelope, and Golgi apparatus [25, 26]; that is, COX-1 isoform subcellular localization is not mainly cytosolic, and COX-1 is embedded in these membranes. Licochalcone A could inhibit collagen-aggregation after a brief period of incubation such as 15 s. Even though precise COX-1 localization in platelets has not been clarified, we can suppose from this result that licochalcones are not completely incorporated into platelets, but approach COX-1 that is located in neighboring platelet plasma membranes and does not allow arachidonic acid to reach the active site of COX-1. Otherwise, the composition of the lipid bilayers of platelet plasma membranes may differ from that of other cells, and licochalcones are easily incorporated into intracellular regions.

Using LC-MS/MS system for PG quantitation, we determined the IC_50_ values of licochalcone A *in vitro* for COX-1 and COX-2 to be 0.94 μM and 1.93 μM, respectively (Fig 6A), and the ratio of IC_50_ of COX-1/COX-2 as being 0.49. Hence, licochalcone A is not a selective inhibitor for these COX isoforms compared with a COX-2-selective inhibitor such as celecoxib, the ratio of which is approximately 9.49 for ovine COX-1/ovine COX-2 and 600 for ovine COX-1/human COX-2 [17]. However, Song et al. showed that licochalcone A did not cause inhibition of COX-2 activity in human keratinocytes [27], whereas we demonstrated that licochalcone A clearly blocked both COX isoforms in a concentration-dependent manner. The discrepancy between these results cannot be explained so far.

In addition, we determined the reaction rate of COX-1 and analyzed the inhibitory mechanism of licochalcone A by enzyme kinetics. The previously reported Km value of COX-1 was in the range of 0.75–4.67 μM [17, 28, 29], which is comparable with our value of 0.6236 μM (Fig 6B). In addition, the Vmax values were comparable regardless of licochalcone A, suggesting licochalcone A causes competitive inhibition of the COX-1 reaction with arachidonic acid. However, in the presence of licochalcone A, COX-1 activity was strongly attenuated when the concentration of arachidonic acid was low (Fig 6B), which was not applied to the Michaelis–Menten equation but rather fitted to a sigmoid curve. It is not easy to interpret this result, but we assume that COX-1 conformation was changed and it lost affinity to arachidonic acid by binding to licochalcone A. However, higher concentrations of arachidonic acid could compete with licochalcone A and recover the affinity to COX-1, resulting in a concentration-dependent sigmoid curve with equivalent Vmax values.

Licochalcones contain an α,β-unsaturated ketone structure, and nucleophiles such as the thiol group of cysteine can bind to licochalcones easily in physiological conditions by the Michael addition reaction. In fact, we could observe covalent binding between licochalcone A and cysteine, and it has been shown that Cys179 in IKKβ is an important amino acid residue for inhibition of IKKβ-mediated nuclear factor-κB activation by licochalcone A [20]. Furthermore, a reduced licochalcone A that lacks a double bond failed to inhibit IKK-mediated nuclear factor-κB activation [30]. These reports suggest the possibility that licochalcone A binds to the cysteine residues of various proteins by the Michael addition reaction. Because COX-1 has a free cysteine residue (Cys512) near the active site [31], we speculated that licochalcones bound to COX-1 at this Cys512, and thus attempted detection of licochalcone-bound COX-1 by mass spectrometry. However, we failed to prove our hypothesis. The interaction between licochalcones and COX-1 may be unstable and reversible as high concentrations of arachidonic acid can replace it (Fig 6B). Further study is necessary to identify the binding site of licochalcones on COX-1 and COX-2.

## Conclusions

In conclusion, we found that licochalcone A isolated from licorice species *Glycyrrhiza inflata* suppressed collagen-induced platelet aggregation by inhibiting COX-1 isoforms. Further study is necessary to clarify the detailed mechanism of COX inhibition by licochalcone A and another potential mechanism of inhibition by other platelet activators including thromboxane A_2_.

## Supporting information

S1 Fig Effect of licochalcone A on ADP-induced platelet aggregation. Licochalcone A (Lico. A, 10 or 100 μM) or DMSO (-) was preincubated for 5 min before addition of ADP (10 μM) in the presence of 1 mM CaCl_2_. Results are shown as mean±S.E.M. (*P<0.05 compared with control, n = 4–7, Dunnett’s method).
(TIF)
