# Supplementary figures and images for: Licochalcones extracted from Glycyrrhiza inflata inhibit platelet aggregation accompanied by inhibition of COX-1 activity
Source: PLoS One. 2017 Mar 10;12(3):e0173628. doi: 10.1371/journal.pone.0173628 (PMC5345862; doi:10.1371/journal.pone.0173628)

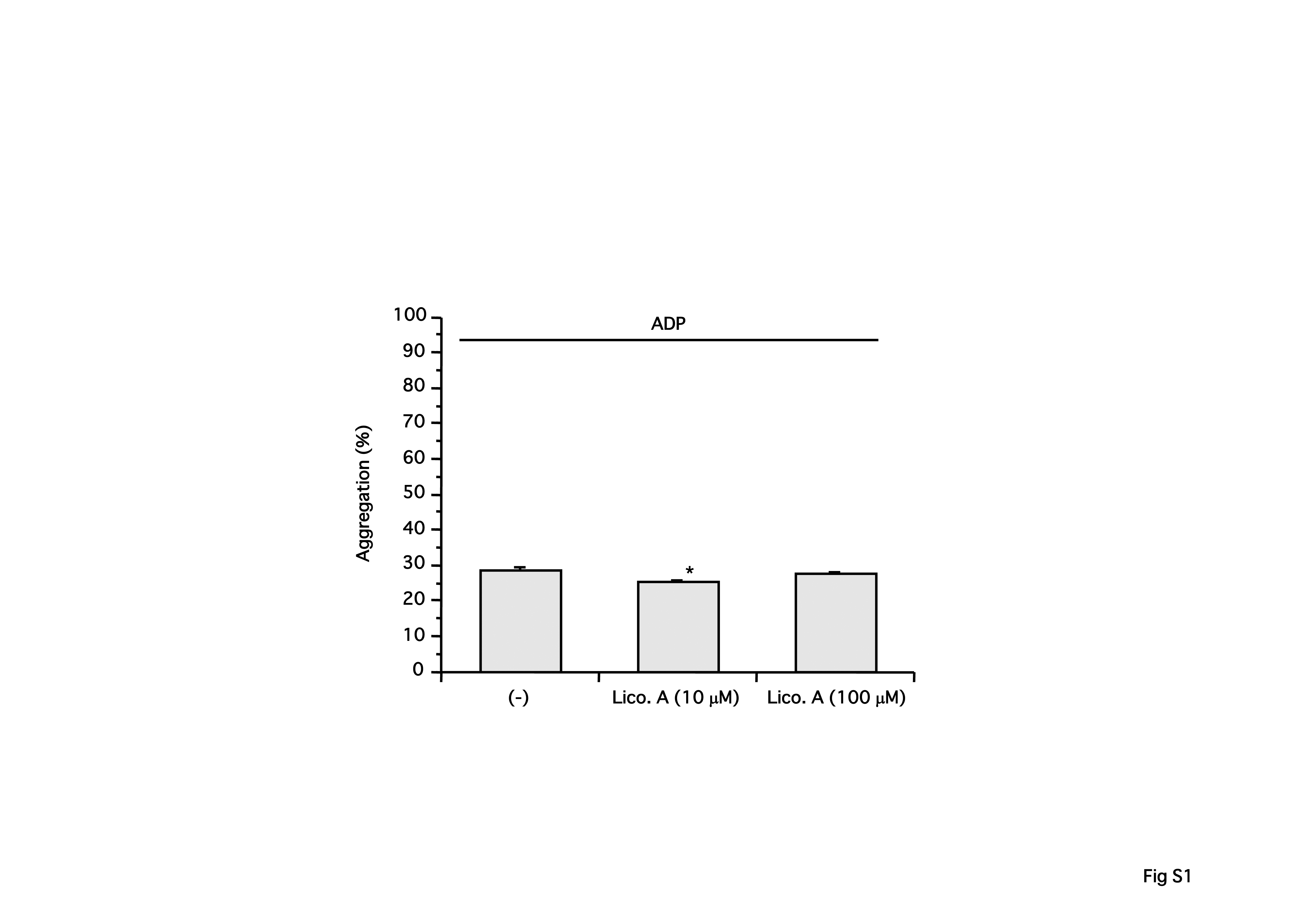

Supplement: S1 Fig — Licochalcone A (Lico. A, 10 or 100 μM) or DMSO (-) was preincubated for 5 min before addition of ADP (10 μM) in the presence of 1 mM CaCl2. Results are shown as mean±S.E.M. (*P<0.05 compared with control, n = 4–7, Dunnett’s method). (TIF) [file pone.0173628.s001.tif]
